# Expression of Measles Virus Nucleoprotein Induces Apoptosis and Modulates Diverse Functional Proteins in Cultured Mammalian Cells

**DOI:** 10.1371/journal.pone.0018765

**Published:** 2011-04-14

**Authors:** Ashima Bhaskar, Jyoti Bala, Akhil Varshney, Pramod Yadava

**Affiliations:** Applied Molecular Biology Laboratory, School of Life Sciences, Jawaharlal Nehru University, New Delhi, India; University of North Carolina at Chapel Hill, United States of America

## Abstract

**Background:**

Measles virus nucleoprotein (N) encapsidates the viral RNA, protects it from endonucleases and forms a virus specific template for transcription and replication. It is the most abundant protein during viral infection. Its C-terminal domain is intrinsically disordered imparting it the flexibility to interact with several cellular and viral partners.

**Principal Findings:**

In this study, we demonstrate that expression of N within mammalian cells resulted in morphological transitions, nuclear condensation, DNA fragmentation and activation of Caspase 3 eventuating into apoptosis. The rapid generation of intracellular reactive oxygen species (ROS) was involved in the mechanism of cell death. Addition of ascorbic acid (AA) or inhibitor of caspase-3 in the extracellular medium partially reversed N induced apoptosis. We also studied the protein profile of cells expressing N protein. MS analysis revealed the differential expression of 25 proteins out of which 11 proteins were up regulated while 14 show signs of down regulation upon N expression. 2DE results were validated by real time and semi quantitative RT-PCR analysis.

**Conclusion:**

These results show the pro-apoptotic effects of N indicating its possible development as an apoptogenic tool. Our 2DE results present prima facie evidence that the MV nucleoprotein interacts with or causes differential expression of a wide range of cellular factors. At this stage it is not clear as to what the adaptive response of the host cell is and what reflects a strategic modulation exerted by the virus.

## Introduction

Measles virus nucleoprotein (N) packages the nonsegmented, negative-sense, single-stranded RNA genome of measles virus to form the helical nucleocapsid [1], [2], [3]. N is divided into two regions: a well-conserved, N-terminal moiety (aa 1–400), and a hypervariable, C-terminal moiety (aa 401–525) [4]. Sequence variability in the N protein C terminus reflects that this protein domain is intrinsically disordered [5], imparting structural plasticity that allows it to mediate interactions with a variety of cellular and viral binding partners that include the viral P protein [6]–[8], the Hsp-72 [9], the cellular nucleoprotein receptor [10], interferon-responsive factor 3 [11], and p40 subunit of eukaryotic initiation factor 3 [12]. N also has the capacity to self-assemble on cellular RNA to form nucleocapsid-like particles in the absence of viral RNA and of any other viral protein [13]–[17].

In the present study, we studied the response of mammalian cells to N expression. Human cell lines MCF7 (breast cancer cells) and 293T (embryonic kidney cells) were used as model system. Cells expressing N underwent apoptosis. There are signs of reactive oxygen species (ROS) building up in cells expressing N. Pretreatment of ascorbic acid (AA) partially counteracts ROS generation and apoptosis. We show that N triggers apoptosis through generation of ROS and caspase-3 activation.

We also carried out a differential profiling of proteins from N expressing cells and control cells using two-dimensional gel electrophoresis (2-DE) followed by matrix-assisted laser desorption/ionization time of flight mass spectrometry (MALDI-TOF-MS). Since N induces apoptosis through building up of ROS, we would expect a proteomics approach to reflect on the associated changes. In this experiment, we not only identified proteins involved in apoptosis and ROS management, but we also identified a number of novel alterations to the cellular proteome. The altered expression of proteins reflected in 2D gels was validated by RT-PCR analysis of 4 proteins and a network of differentially expressed proteins was established. While the results presented here are expected to offer some clues to further study the viral infection mechanisms, we see a new trigger to induce cell death in the measles virus nucleoprotein which is amenable to re-engineering as a targeted molecule.

## Results

### Expression of N results in morphological transitions and appearance of hypodiploid nuclei in MCF7 cells

**Figure 1:**
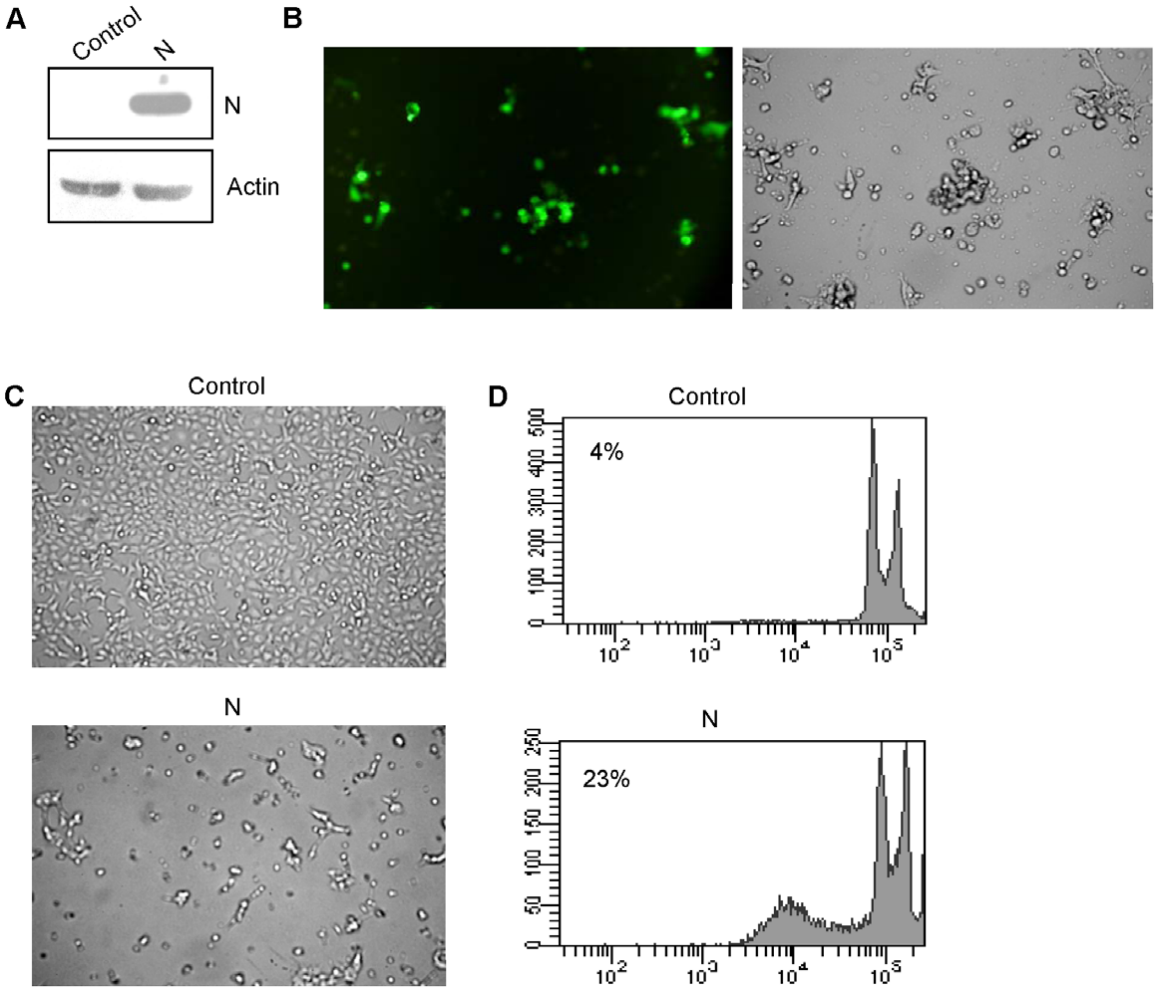
N induces cell death in MCF7 cells. (a) Immunoblot analysis of N expression in MCF7 cells. (b) Representative fluorescent images of cells co transfected with GFP and N expression vectors showing 70–80% transfection efficiency. (c) Morphological features of MCF7 cells expressing N as seen by light microscopy. (d) Flow cytometric profile of representative cell populations 24 h post transfection, fixed and PI stained for cell cycle analysis as described in methods. The data is representative of three independent experiments.

To investigate the effect of N in mammalian cells, we used the breast cancer cell line MCF7 as a model. The expression of N protein was checked by western blotting 24 h after transfection of pCA-N in MCF7 cells. A 60 kD band corresponding to N was clearly visible in N transfected cells (Fig. 1a). Transfection efficiency was checked by co transfecting GFP and N expression vectors. Approximately 80% of the cells showed fluorescence (Fig. 1b). We observed by microscopy that N induced morphological features typical of apoptosis. Cells expressing N appeared enlarged, round, dispersed and damaged compared with control cells. Some cells appeared detached displaying apoptotic morphology (Fig. 1c). Increased undulations on surface of cells expressing N protein were indicative of apoptosis.

Next we analyzed the typical apoptosis-associated leakage of fragmented DNA from apoptotic nuclei by the Nicoletti method [18] and subdiploid fraction (characteristic of apoptosis) by flow cytometry. Cells were transfected with N expression vector and 24 h post transfection, cell cycle analysis was done. There was an increase in hypodiploid sub-G_0_ fraction comprising both apoptotic and ghost cell population, implying together the extent of cell death (Fig. 1d). The results indicated that, compared with the control, N expressing cells had larger portion of cells in sub-G_0_ phase (23% as against 4%) (Fig. 1d).

### N induced cell death involves ROS generation and activation of caspase-3

**Figure 2:**
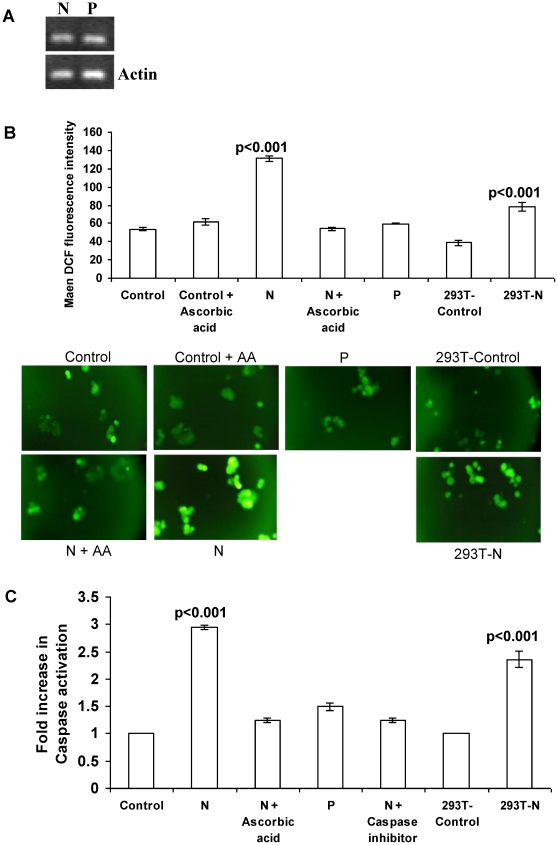
N induces ROS production and caspase 3 activation in MCF7 and 293T cells. (a) N and P expression in MCF-7 cells. 24 h after transfection, total RNA was extracted from the cells and RT-PCR was performed as described in methods. Gel pictures showing expression of N and P. (b) Analysis of ROS generation by DCF fluorescence as described in Methods. Representative fluorescent images and corresponding mean fluorescence intensity. (c) Determination of caspase 3 activation in MCF7 cells by FITC fluorescence as described in Methods. Results are expressed as the fold increase in fluorescence and are given as the mean ± SD for three experiments.

To determine whether ROS is involved in N induced apoptosis, concentration of ROS in N expressing cells was analyzed by fluorescence of oxidized 2′, 7′-dichlorofluorescein in an ELISA reader. There was a 2.9 fold increase in concentration of ROS in cells expressing N (Fig. 2b) compared to control cells (p<0.001). Change in ROS induced by N expression was inhibited by AA to a level that was almost as low as in control cells (Fig. 2b). As a negative control, MCF7 cells transfected with measles virus phosphoprotein (P) were also checked for ROS generation. To check for N and P expression, RT-PCR was performed. N expression was comparable to that of P (Fig. 2a). In P expressing MCF7 cells there was insignificant increase in ROS levels when compared to control (Fig. 2b).

The activation of caspase-3 is a central event in the process of apoptosis [19]. To evaluate further the mechanism of N induced cell death in MCF7 cells, the activation of the caspase-3 protein was examined. N expression in MCF7 cells resulted in a 3-fold increase (p<0.001) in activation of caspase-3 (Fig. 2c) compared to control cells whereas no significant activation of caspase-3 was seen in P expressing MCF7 cells (Fig. 2b) Caspase activation was inhibited to the level of control cells by the caspase family inhibitor Z-VAD-FMK and by pretreatment of N transfected cells with AA (Fig. 2c).

**Figure 3:**
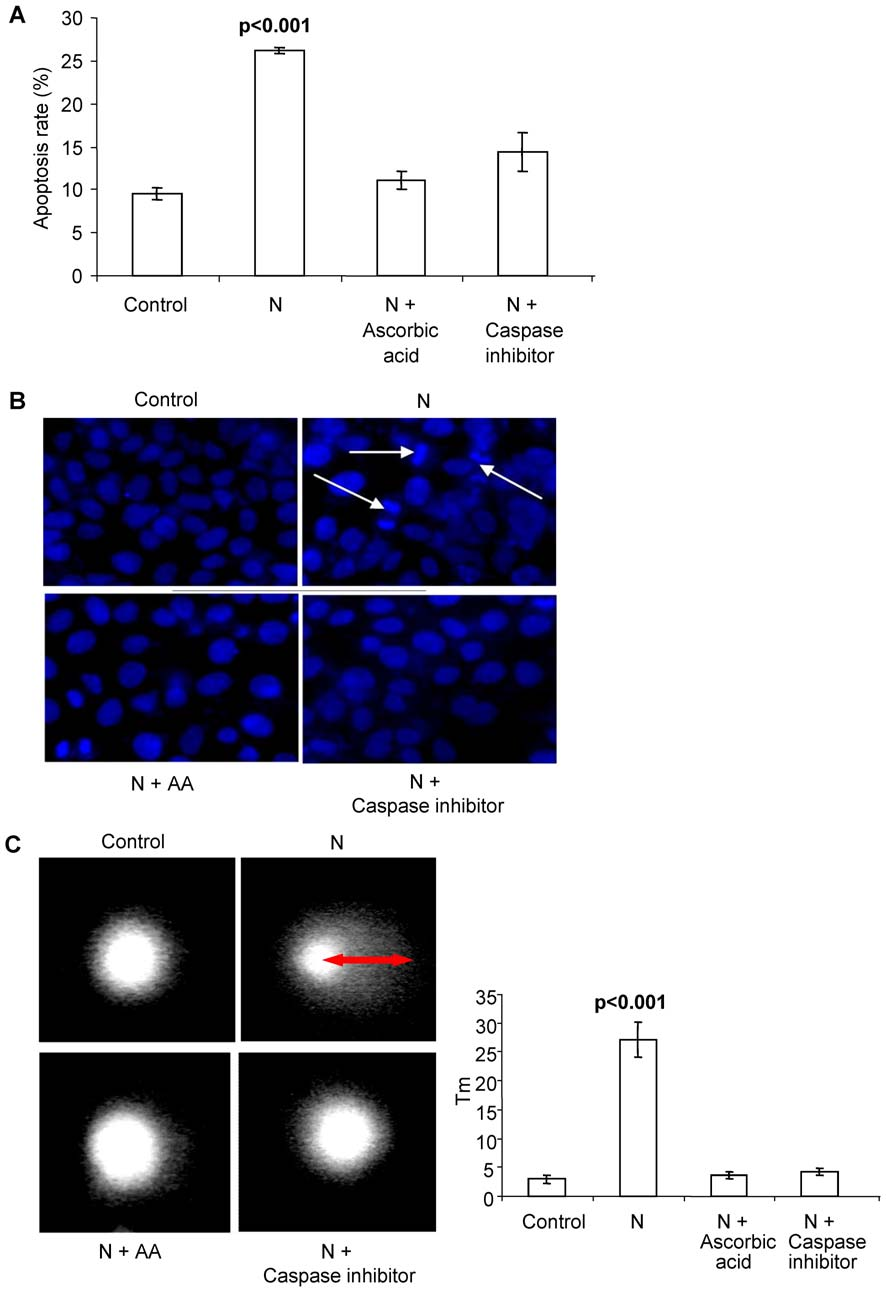
Inhibition of apoptosis by AA and Z-VAD-FMK. (a) Percentage of apoptosis in control N expressing cells analyzed by flow cytometry after PI staining. Apoptotic cells were estimated by the percentage of cells in the sub-G1 peak. Each bar represents the mean ± SD of three independent experiments. (b) Nuclear staining with hoechst 33258. N transfected MCF7 cells showed apoptotic morphology; DNA condensation and nuclear fragmentation whereas rest of the cells remained uniformly stained with round and unpunctuated nucleus. White arrows indicate nuclear condensation and fragmentation. The figures represent one of the three independent experiments. (c) Single Cell Gel Electrophoresis assessment of N toxicity in human breast cancer cells (MCF7). Cells were harvested for comet tail formation assays under alkaline conditions. Comet images were captured using fluorescence microscopy, and tail moment was analyzed in 50–60 randomly chosen comets using Comet assay IV software. Representative comet images observed are shown. Histograms represent changes in the comet tail moments between control and treated cells. N transfected cells show a long tail indicating DNA damage. Each experiment was done in triplicate. Data is represented as means ±SD.

To further confirm the role of ROS and involvement of caspase 3, apoptosis was checked in N transfected cells pretreated with the antioxidant AA or caspase 3 inhibitor Z-VAD-FMK by propidium iodide (PI) staining (Fig. 3a). Both treatments inhibited N-induced apoptosis. Hypodiploid fraction in case of the N-transfected cells pretreated with AA (11.1%) and treated with Z-VAD-FMK (9.8%) was comparable to control cells (9.6%) whereas it was 26.1% in case of cells expressing N (p<0.001) (Fig. 3a).

DNA fragmentation is a hallmark of apoptotic cells [20]. To examine the nuclear morphological changes in response to N expression, MCF7 cells were stained with the fluorescent dye Hoechst 33528 and visualized under a fluorescence microscope. The control cells remained uniformly stained whereas N expressing cells exhibited nuclear condensation and DNA fragmentation (Fig. 3b). N transfected MCF7 cells pretreated with AA or Z-VAD-FMK also remained uniformly stained and showed negligible signs of nuclear condensation (Fig. 3b).

Comet assay was also used to assess the differences in DNA damage accumulation between control and N expressing MCF7 cells. The concept underlying the comet assay is that undamaged DNA retains a highly organized association with matrix proteins in the nucleus. When damaged, this organization is disrupted. The individual strands of DNA lose their compact structure and relax, expanding out of the cavity into the agarose. When the electric field is applied to the DNA, it is drawn towards the anode. Undamaged DNA strands are too large and do not leave the cavity, whereas the smaller the fragments, the farther they are free to move in a given period of time. Therefore, the amount of DNA that leaves the cavity is a measure of the amount of DNA damage in the cell. The brighter and longer the tail, the higher is the level of damage. N expression induced statistically significant increase in tail moment (27.07, p<0.001) indicating significant increase in DNA damage when compared with control (3.03) (Fig. 3c). Treatment of N transfected cells with AA or Z-VAD-FMK reduced the tail moment to 3.62 and 3.21 respectively (Fig. 3c).

Apoptotic effects of N were also studied on the non cancerous human embryonic kidney cell line 293T in terms of ROS generation and caspase-3 activation. 24 h post transfection there was a 2 fold increase in ROS concentration in N transfected 293T cells as compared to mock transfected 293T cells (p<0.001) (Fig. 2a). Similarly, N expression resulted in 2.4 fold increase in caspase-3 activation when compared with mock transfected cells (p<0.001) (Fig. 2b).

### Two-Dimensional Gel Electrophoresis (2DE)

**Figure 4:**
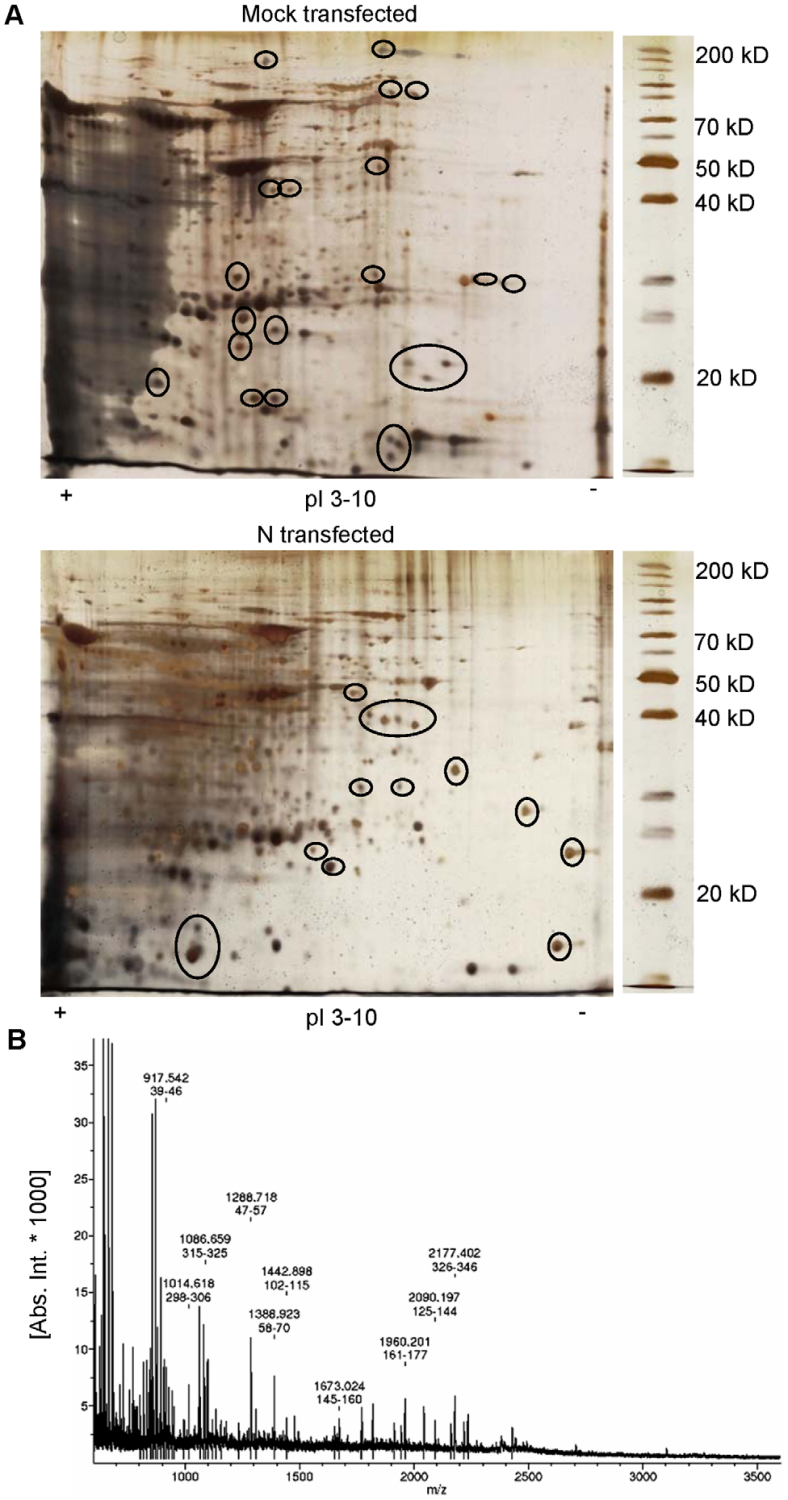
Typical 2D gel analysis of N transfected cells compared with control cells. Cells were transfected with either pCA or pCA-N and 24 h post transfection total cellular protein was extracted. Protein extracts were subjected to separation by 2DE prior to staining and visualization as described under methods. (a) Representative 2D gels of control and treated samples. Encircled proteins were consistently seen to vary in intensity in multiple experiments. Those spots were picked for sequencing. (b) The MALDI-TOF-MS mass spectrum of a spot, identified as the Poly(rC) binding protein 1 according to the matched peaks is shown.

We performed 2DE proteomic analysis to evaluate the differences in protein expression between control and N transfected cells (24 h), in which the first dimension was fractionated over an IEF range of pH 3 to 10 and molecular mass was separated in 12% polyacrylamide gel. The 2DE gels were highly reproducible and image analysis performed on triplicate 2DE gels produced minimal inter-sample and intra-sample variation in terms of normalized volumes of cellular proteins and proteome profile. Representative silver stained gels from three independent experiments are shown in Fig. 4a, where some differentially expressed proteins are encircled. Differentially expressed proteins were detected using ImageMaster™ 2D Platinum 5.0 software.

### Mass spectrometric analysis

All protein spots identified as having significant changes in expression levels and present in sufficient amounts to be visible on the gel were excised for mass spectrometric analysis. A total of 25 proteins with significantly differential expression levels were identified by mass spectrometry (Table 1, 2). Of the 25 identified proteins, 11 proteins were up-regulated in cells expressing N (Table 1), whereas 14 proteins were down-regulated as a result of N expression (Table 2). The altered proteins were classified in terms of their subcellular location and biological function by information from Swiss-Prot. The majority of the proteins were located in the cytoplasm (11), with fewer in the nucleus (5). Of the remaining proteins, 3 were cytoskeletal proteins, 3 located in the cell membrane, 1 in mitochondrion and 1 extracellular. The differentially expressed proteins are involved in diverse biological functions like cell structure (1↑,1↓), signal transduction (1↑,2↓), metabolic processes (1↑,2↓), gene expression (3↑,3↓), redox maintanence (2↑,1↓), chaperons (1↑,1↓) and others (2↑,4↓). Three of the differentially expressed proteins are known to be pro-apoptotic (2↑,1↓) and 8 are anti-apoptotic (4↑,4↓).

**Table 1:** List of proteins positively associated with N expression as identified by MALDI-TOF analysis.

Protein	Biological process	Mw/pI	Score	Sequence Coverage (%)	Localization
Cofilin 1 (CFL1)	Rho protein signal transduction	17/9.3	61	47	Cytoskeleton
Eukaryotic translation initiation factor 5A (eIF5A)	protein biosynthesis	17/4.9	40	31	Nucleus
Glutathione S-transferase (GST)	glutathione transferase activity	23.5/5.3	109	59	Cytoplasm
Glyceraldehyde-3-phosphate dehydrogenase (GAPDH)	glycolysis	36.5/9.3	59	30	Cytoplasm
HLA-C	antigen presentation	38/5.7	58	35	Cell membrane
Parkinson disease protein 7 (PARK7)	chaperone	20/6.4	61	59	Cytoplasm
Peroxiredoxin 3 (PRDX3)	cell redox homeostasis	24.9/5.7	41	17	Cytoplasm
Phosphatidylethanolamine-binding protein (PEBP1)	protease inhibitor	21/5.4	51	43	Cytoplasm
Poly(rC) binding protein 1 (PCBP1)	RNA splicing	38/6.8	93	40	Nucleus
Proliferation-associated 2G4 (PA2G4)	transcription and translation regulation	41.2/6	55	22	Nucleus
Rhomboid like protein 2	signal transduction	142/7	42	21	Cell membrane

Score and sequence coverage were generated by MASCOT software. All identifications were confirmed using MASCOT MS/MS ion search and/or peptide-fingerprinting and significant matches (p<0.05) were retained.

**Table 2:** List of proteins negatively associated with N expression as identified by MALDI-TOF analysis.

Protein	Biological process	Mw/pI	Score	Sequence Coverage (%)	Localization
Actin filament-binding protein frabin (FGD4)	actin cytoskeleton organization	86/5.8	44	22	Cytoskeleton
Diacylglycerol lipase beta (DAGLB)	lipid catabolic process	25/9.5	61	31	Cell membrane
Heat shock 70 kDa protein 1 (HSPA1A)	stress response	70/5.4	147	45	Cytoplasm
Lysozyme C (LYZ)	cytolysis	147/9.2	63	52	Extracellular
Mitogen-activated protein kinase kinase 4 (MAP2K4)	protein amino acid phosphorylation	44/8.3	50	50	Cytoplasm
MAP4K4	protein amino acid phosphorylation	142/7	51	45	Cytoplasm
Myosin IIIB (MYOIIIB)	sensory/vision	151/8.3	52	33	Cytoskeleton
Nucleoside diphosphate kinase A (NME1)	nucleotide metabolism	17.3/5.8	45	32	Nucleus
Peroxiredoxin 2 (PRDX2)	cell redox homeostasis	22/5.6	93	38	Cytoplasm
Phosphoglycerate dehydrogenase (PHGDH)	amino-acid biosynthesis	57.4/6.3	47	16	Cytoplasm
Probable global transcription activator SNF2L1 (SMARCA1)	transcription regulation	117.9/8.6	45	10	Nucleus
Prohibitin (PHB)	DNA synthesis	29.8/5.5	77	49	Mitochondrion
Protein FAM124A	uncharacterized	51.6/7.1	52	11	Uncharacterized
Serpin peptidase inhibitor (SERPINB6)	protease inhibitor	44.3/5.2	50	11.5	Cytoplasm

Score and sequence coverage were generated by MASCOT software. All identifications were confirmed using MASCOT MS/MS ion search and/or peptide-fingerprinting and significant matches (p<0.05) were retained.

### Analysis of differential protein expression using semi quantitative and real time quantitative RT-PCR

**Figure 5:**
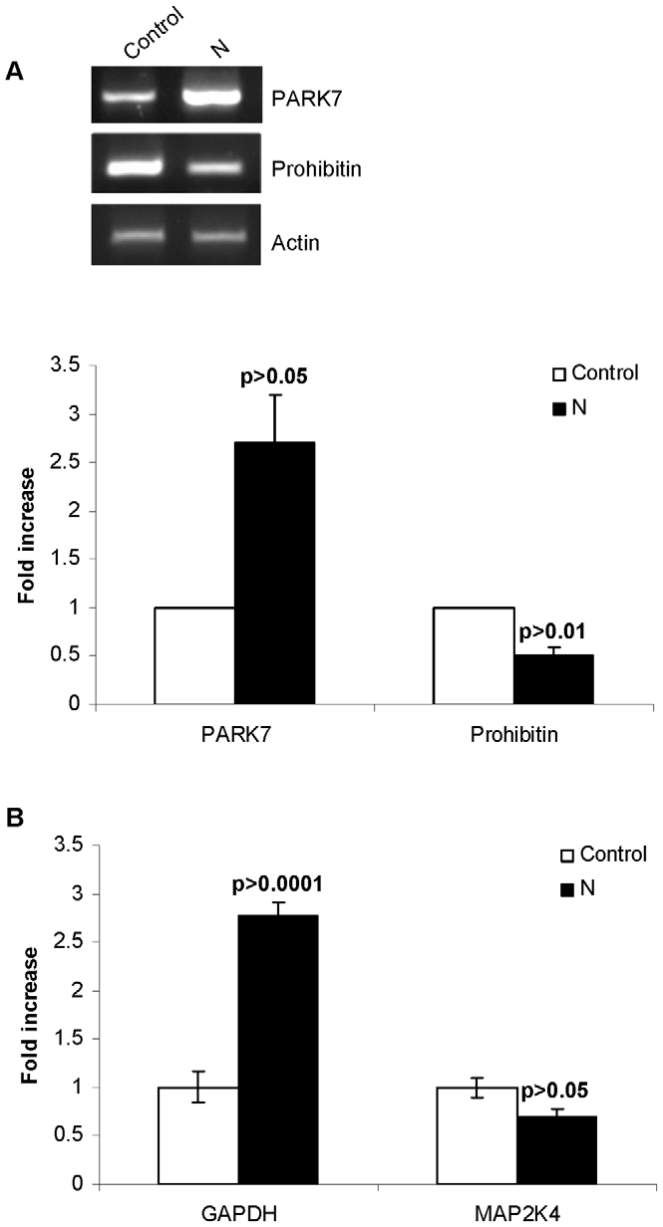
Validation of 2DE by real time and semi quantitative RT-PCR. 24 h after transfection, total RNA was extracted from the cells and real time and semi quantitative RT-PCR was performed as described in methods. (a) Gel pictures showing differential expression of PARK7 and PHB. Fold change in expression was analyzed by software ImageJ. Data was normalized with beta actin as the control house keeping gene. (b) Relative quantification of GAPDH and MAP2K4 mRNA in N transfected and control MCF7 cells. The change in gene expression is expressed as fold change in relation to control. Results are presented as the mean ± SD from 3 different experiments.

To validate 2DE results, we performed relative quantification real-time PCR and semi quantitative RT-PCR on 4 genes representative of housekeeping metabolism, cell cycle control and pathological states. Expression levels of GAPDH and MAP2K4 were detected by real time quantitative RT-PCR analysis whereas PHB and PARK7 transcripts were checked by semi quantitative RT-PCR. In general, the trends in the change in mRNA abundance of these genes were similar to the change patterns of corresponding proteins in 2-DE gels. Semi quantitative results showed that transcripts of PARK7 were increased by 2.7 fold (p<0.05) in N expressing cells as compared to control cells whereas the transcripts of PHB were significantly decreased by 2 fold (p<0.01) in cells expressing N as compared to control cells (Fig. 5a). The real-time PCR showed up-regulation of GAPDH mRNA levels and down-regulation of MAP2K4 mRNA levels in N expressing cells when normalized with the internal control, beta actin (Fig. 5b). The mean ± SD relative expression of GAPDH mRNA in N expressing cells (2.76±0.14, p<0.0001) was significantly higher than in control cells (1±0.16). Similarly, the mean ± SD relative expression of MAP2K4 mRNA in N expressing cells (0.7±0.07, p<0.05) was significantly lower than in control cells (1±0.1).

### Protein network

**Figure 6:**
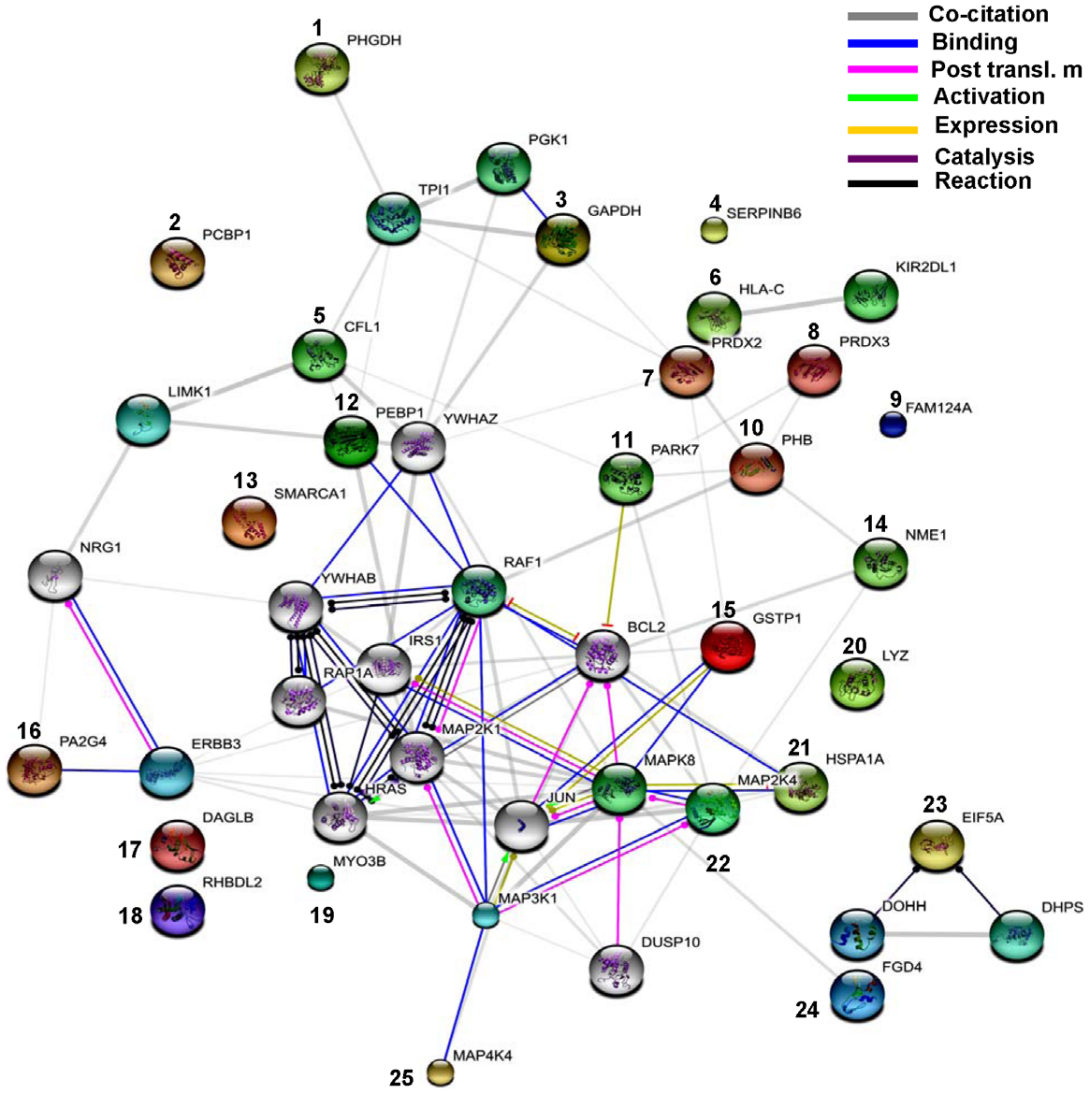
Protein interaction map. Map was prepared using the STRING web tool (http://string.embl-heidelberg.de/) using default parameters and the accession for identified proteins. Different types of interactions are depicted by different colored lines. The legend of the interaction network is summarized in the figure. Numbers show the identified proteins. These proteins interact with each other as well as with some other predicted functional proteins.

To determine possible functional cross-talk among differentially expressed target proteins, we built a protein network with String software (Fig. 6). Of the 25 unique identified proteins, 15 showed connectivity with at least one other protein in the map. Proteins involved in redox maintenance clustered together (PARK7, PRDX2, PRDX3, PHB, GSTP1). Cross talk was seen between apoptotic proteins and antioxidants (for instance PARK7 a redox sensing and antioxidant protein interacts with the anti-apoptotic protein BCL2). Some other predicted functional partners include Deoxyhypusine hydroxylase (DOHH), Receptor tyrosine-protein kinase erbB-3 precursor (ERBB3), Mitogen-activated protein kinase kinase kinase 1 (MAP3K1), LIM domain kinase 1 (LIMK1), Deoxyhypusine synthase (DHPS), Triosephosphate isomerase (TPI1), RAF proto-oncogene serine/threonine-protein kinase (RAF1), Phosphoglycerate kinase 1 (PGK1), Mitogen-activated protein kinase 8 (MAPK8), Killer cell immunoglobulin-like receptor 3DL2 precursor (KIR2DL1), etc. 8 out of 10 remaining proteins did not show interaction with any other cellular protein.

## Discussion

Apoptosis or programmed cell death involves a series of biochemical events that lead to a variety of morphological changes, including blebbing, changes to the cell membrane such as loss of membrane asymmetry and attachment, cell shrinkage, nuclear fragmentation, chromatin condensation, and chromosomal DNA fragmentation [21]. Defective apoptosis is the major causative factor in the development and progression of cancer. Therefore, a great importance is given to agents known to induce apoptosis in cancer cells.

In the present study, we investigated the pro apoptotic effects of N in human breast cancer cells. Various morphological changes characteristic of apoptosis, such as cell rounding, detachment, shrinkage and nuclear condensation were observed in cells expressing N. The most convenient system for the analysis of apoptosis is visualization of hypodiploid nuclei after PI staining and flow cytometry. Cells with hypodiploid content of nuclear DNA did appear following N expression. DNA fragmentation was also detected by comet assay which is a highly sensitive method for assessing DNA damage at the individual cell level [22]. Our *in vitro* results demonstrate that N induces apoptosis in ROS dependent manner and involves activation of caspase-3.

Nucleoprotein is the most abundant of the measles virus proteins expressed during infection and carried along with the virion. It gets exposed to the intracellular proteins during virus proliferation and is likely to interact with many of them to exert regulatory or direct functional influences in order to maximize progression of the virus. It mediates the immune response, induces immunological abnormalities and influences pathogenicity during the virus life cycle. To further investigate the possible actions of N during viral infection, we performed 2DE to study the proteome changes that occurred in MCF7 cells following N expression. Our 2DE results present a prima facie evidence that the MV nucleoprotein indeed interacts with or cause differential expression of a wide range of cellular factors that are (a) located on cell surface e.g., DAGLB, HLA-C and RHBDL2, (b) secreted extracellularly e.g., LYZ, (c) remaining in cytoplasm e.g., GST, GAPDH, Hsp70, MAP kinases, PARK7, PRDXs, PEBP1, PHGDH, and SERPINB6, (d) forming cytoskeletal elements e.g., FGD4, CFL1, MYOIIIB, (e) associated with mitochondria e.g., PHB or (f) nuclear e.g., NME1, PCBP1, SMARCA1, and PA2G4. The nature of the effects is variable with 11 out of 25 differentially expressed proteins showing signs of over-expression while 14 show down-regulation in the presence of N. 2DE results were validated by real time and semi quantitative RT-PCR analysis of 4 proteins. We selected these four proteins to represent diverse functional classes viz. housekeeping metabolism, (GAPDH), cell cycle control (MAP kinases), ROS management and pathological links (PARK7) and DNA synthesis (PHB). At this stage it is not clear as to what the adaptive response of the host cell is and what reflects a strategic modulation exerted by the virus.

Since we have shown that N induces apoptosis through generation of ROS, we found the change in expression level of many proteins involved in ROS management and apoptosis These included Cofilin, eIF5A, GST, GAPDH, PRDXs, Hsp70, PARK7, PHB and PA2G4. Cofilin (↑) which is an intracellular actin-modulating protein involved in vesicular trafficking, is involved in oxidant-induced apoptosis [23], [24]. It is also known that interaction of measles virus glycoprotein complex with T cells results in reduced phosphorylation levels of cofilin [25]. Since we notice an augmented expression of cofilin in cells expressing N, it will be interesting to see if the N-induced effects on cofilin are also associated with altered phosphorylation of the protein and if effects of N and glycoprotein complex are in reciprocal directions. Recent studies indicate that eIF5A (↑) may also play a role in cell death [26], [27]. GSTs (↑) are a superfamily of enzymes that mainly catalyze the conjugation of glutathione to a wide range of exogenous and endogenous electrophilic substrates, including chemical carcinogens, therapeutic drugs and oxidative stress products.

GAPDH (↑), in addition to its long established metabolic function, has recently been implicated in several non-metabolic processes, including transcription activation [28], initiation of apoptosis [29], [30], and reversible metabolic switch under oxidative stress [31]. PRDXs (↑, ↓) control the constitutive levels of H_2_O_2_ in the cell and protect against ROS-induced damage by catalyzing the reduction of the H_2_O_2_ into water. While gain in glyceraldehyde phosphate dehydrogenase and peroxiredase in cells can correlate with enhanced metabolic rate, an apparent loss of redox management enzymes can result in apoptosis. The Hsp70 (↓) is an important part of the cell's machinery for protein folding, and helps to protect cells from stress. It is expressed in most of the cancer cells [32] and inhibits apoptosis [33]. PARK7 (↑) may function as a redox-sensitive chaperone and as a sensor for oxidative stress. It promotes cell survival by protecting cells from oxidative stress [34], [35]. Since it is also associated with Parkinson's disease, it will be important to see if there is any mechanistic relationship between neuronal disorders caused by persistent infection with measles virus and its apparent correlation with PARK7 over-expression. PHB (↓) acts as a chaperone for respiration chain proteins. It represses the activity of E2F1 transcription factor [36], [37] which has the ability to induce apoptosis [38]. Down regulation of anti-proliferative factors like PHB and gain in proliferation associated factor PA2G4 may be part of cellular countercurrents of signaling in the situation of stress caused by virus infection.

Down regulation of the secretary lysozyme C may be a strategy to prevent stimulation of virus infected cells by cells wall constituents like LPS of bacteria released following muramidase action. Possible interactions of MAP kinases (↓) with measles virus replication machinery remain to be investigated. Similarly the role of global transcription factors such as SMARCA1 (↓), likely to be modulating chromatin organization, is not well understood. Down regulation of the serine protease inhibitors (SERPINB6) would favor endoprotease-mediated processing of viral proteins. A genetically engineered serpin was shown to inhibit the production of infectious MV due to non-maturation of precursor of MV-F via proteolytic cleavage [39]. DAGLB catalyzes a key step in arachidonate pathway and its down regulation in the presence of N is likely to have a bearing on the organization and integrity of the cell wall.

To summarize our results, we may conclude that N induces the death of mammalian cells which involves increase in ROS generation and procaspase-3 activation. Using proteomic analysis after N expression, we identified 10 up/down-regulated proteins involved in ROS and apoptosis. By reengineering N protein, we can use it as a targeted therapeutic against cancer cells. It might be possible to use gene delivery vehicles to specifically target N towards diseased cells. For instance, modified adenovirus vectors capable of entering cells via specific receptors other then coxsackie adenovirus receptor could be used for delivering N gene to cancer cells [40], [41]. We also observed proteome changes related to varied cellular functions. It must be underlined that the present study used expression of nucleoprotein in isolation from other viral proteins and the associated changes may be at variance from the scenario of real infection process. The list of proteins showing differential expression is by no means a comprehensive one and further studies on the subject would be required to establish a resolved picture of proteomic alterations and their possible functional relevance.

## Materials and Methods

### Cell Culture and Transient Transfections

MCF7 and 293T cells obtained from NCCS, Pune were cultured in DMEM (Sigma) supplemented with 10% FBS (GIBCO), 1% penicillin/streptomycin (Sigma), in 5% CO_2_ in a humidified atmosphere. Plasmid DNA transfections were carried out in 12-well plates (Tarson, India) using LipofectamineTM 2000 Transfection Reagent (Invitrogen) as per manufacturer's instructions. Briefly, day before transfection, 4×10^5^ MCF-7 cells were plated in 1 ml of medium/well. For each well, 2 µl of LipofectamineTM 2000 Transfection Reagent (Invitrogen) was mixed with 1 µg of the plasmid DNA of interest in serum-free Opti-MEM (Invitrogen) to allow the formation of DNA-LipofectamineTM 2000 Transfection Reagent complexes. The complexes were added to the respective wells and mixed by gently rocking the plate back and forth. Cells were incubated in a 5% CO_2_ incubator at 37°C for 24 h before analysis. For N and P expression, pCA-N and pCA-P were used (kind gift from Prof. Martin Billeter). Mock transfections carried out using empty pCA plasmid were taken as control. Efficiency of transfection was assayed in parallel experiments employing GFP as a reporter.

### Western Blotting

Proteins resolved on SDS-PAGE gels were transferred onto a polyvinylidene difluoride membrane (Milipore) by electroblotting. Primary and secondary antibody binding was done according to standard protocols. The primary antibody was a mouse immunoglobulin G recognizing nucleoprotein (kind gift from Prof. Martin Billeter). The secondary antibody was an anti-mouse immunoglobulin G whole antibody conjugated to horseradish peroxidase (Santa Cruz). Antibody binding was detected with the enhanced ChemiLuminesence system (ECL; Amersham Biosciences), and the results were recorded on Kodak XAR photographic film.

### RT-PCR

The expression of PHB and PARK7 was detected by RT-PCR. 24 h post transfection, total RNA was extracted from MCF7 cells using TRI Reagent (Applied Biosystems, Ambion). cDNA synthesis was carried out by Maxime RT-PCR premix kit (iNtRON Biotechnology). For PCR, 5 µl of the cDNA sample was added to a 45 µl PCR mixture. PCR conditions were as follows: 94°C for 30 sec, 60°C for 30 sec, and 72°C for 30 sec. After 30 cycles, equal volumes of the resulting PCR reactions were analyzed by electrophoresis on a 2% agarose gel. The integrated optical density (IOD) was determined for each PCR product using ImageJ software (http://rsbweb.nih.gov/ij/). Beta actin was used as an internal control. The oligonucleotides used are described in Table 3.

**Table 3:** List of oligonucleotides used in this study.

Gene	Forward primer (5′-3′)	Reverse primer (5′-3′)	Annealing temperature	Amplicon size
GAPDH	AGCCTCAAGATCATCAGCAATG	ATGGACTGTGGTCATGAGTCCTT	60°C	111 bp
β Actin	ATGTGGCCGAGGACTTTGATT	AGTGGGGTGGCTTTTAGGATG ′	60°C	107 bp
β Actin	GCTCCGGCATGTGCAA	AGGATCTTCATGAGGTAGT	54°C	542 bp
MAP2K4	TGTGACTTCGGCATCAGTGG	GCGCTTGGGTCTATTCTTTCA	60°C	101 bp
PHB	CAGGTGGCTCAGCAGGAAGC	TGAAGTGATTTTACCTTTATTTCC	60°C	409 bp
PARK7	ATCTGAGTCTGCTGCTGTGA	ACACGATTCTCAGAGTAGGT	60°C	192 bp

### Real-time RT-PCR

GAPDH and MAP2K4 differential expression was confirmed by SYBR Green-based real-time RT-PCR. RNA (1 µg) was reversed transcribed to cDNA and subjected to real time PCR performed using the Applied Biosystems 7500 Fast Real-Time PCR System. The cycling conditions were 95°C for 15 s, 60°C for 1 min, for 40 cycles, followed by a melting point determination or dissociation curves. Melting curve was performed to verify the presence of a single amplicon. Non template control (NTC) was used as a negative control. The expression level of each gene is indicated by the number of cycles needed for the cDNA amplification to reach a threshold and the results are normalized to beta-actin. The oligonucleotides used are described in Table 3.

### Detection of Reactive Oxygen Species

Reactive oxygen species were detected using the probe 2′, 7′-Dichlorofluorescein diacetate (H2DCF-DA; Sigma) which is a nonfluorescent cell-permeable compound. Within the cell, esterases cleave the acetate groups on DCFH-diacetate, thus trapping the reduced probe (DCFH) intracellularly. ROS in the cells oxidize DCFH, yielding the fluorescent product 2′, 7′-dichlorofluorescein DCF. The fluorescent signal detected (excitation, 485 nm; emission, 530 nm) is proportional to ROS production. Cells (1.0×106) harvested 24 h post transfection were incubated for 1 h at 37°C with 10 µmol L^−1^ of DCF-DA, and relative ROS units were determined using a fluorescence ELISA reader. An aliquot of the cell suspension was lysed, the protein concentration was determined, and the results were expressed as arbitrary emission units per mg protein. In the experiments of inhibition of ROS by antioxidant AA, cells were pre-treated with 100 µM AA for 24 h prior to transfection.

### Determination of apoptosis (by FACS)

MCF7 cells were washed twice with cold-phosphate buffered saline (PBS), fixed in cold 80% ethanol for 30 min, stained with PI (25 µg ml^−1^) with simultaneous treatment of RNase at 37°C for 30 min and analyzed using a FACSCalibur flow cytometer (Becton Dickenson, San Jose, CA) CellQuest software (Becton Dickinson) was used to determine the relative DNA content based on the presence of a red fluorescence. A large number of flow cell events (typically 2×10^5^ cells) were pooled to represent each experimental set in order to minimize noise. The fraction of cells with hypodiploid DNA content was considered as the apoptotic cell population.

### Caspase Activation Assay

Caspase-3 activation assay was performed using CaspGLOW Fluorescein Caspase Staining Kit (Biovision) as per the manufacturer's instructions. 24 h post transfection 1.0×10^6^ cells were treated with FITC-VAD-FMK for 1 h at 37°C in an incubator with 5% CO_2_. After washing twice with the wash buffer, cells were resuspended in 100 µl wash buffer, transferred in a microtiter plate and fluorescence intensity was measured at excitation 485 nm and emission 530 nm. In the experiments of inhibition of caspase activation, the caspase family inhibitor Z-VAD-FMK was added at 1 µl ml^−1^ to the induced culture.

### Hoechst 33258 nuclear staining assay

To study the nuclear morphology, nuclear staining with Hoechst 33258 (Sigma) was performed as described elsewhere [42]. Briefly, 24 h post treatment the floating and trypsinized-adherent populations of cells were fixed with 4% paraformaldehyde in PBS, washed, incubated with 0.125 µg ml^−1^ of Hoechst 33258 at room temperature for 30 min and finally smeared onto microscope slides. Nuclear morphology was then examined under a fluorescent microscope (Meiji Techno TC5000).

### Comet assay

DNA damage was assessed using the alkaline single-cell gel electrophoresis assay (comet assay). The protocol used for comet assay followed the guidelines purposed by Tice [43]. N-transfected and control cells (∼1×10^4^ cells in 20 µl) were added to 60 µl of 0.5% low melting point agarose at 37°C, layered onto a slide pre-coated with 1.5% regular agarose, and covered with a coverslip. After brief agarose solidification in refrigerator, slides were immersed in lysis solution for about 1 h. Prior to electrophoresis, the slides were left in alkaline buffer for 20 min and electrophoresed for another 20 min, at 25 V and 300 mA. After electrophoresis, the slides were neutralized in 0.4 M Tris-HCl (pH 7.5) for 15 min, stained with ethidium bromide, fixed in absolute ethanol and analyzed. Cells with damaged DNA displayed high migration of DNA fragments from the nucleus, forming a tail in comet form. DNA migration was analyzed by fluorescence microscopy (Meiji Techno TC5000). The tail moment (tm) was determined using the software “Comet assay IV” (Perceptive Instruments Ltd.). The tm considers the length of the tail as well as the intensity of the fluorescence staining of the tail, compared to the staining of the comet core. From each sample, 50 randomly selected cells (25 cells from each of two duplicate slides) were analyzed.

### 2-Dimensional gel electrophoresis

Cell pellet was washed with PBS, and cells were lysed in 8 M urea, 4% CHAPS, 100 mM DTT, 1 mM PMSF, 1% protease inhibitor cocktail and 2% pharmalyte ((GE Healthcare)). A 13 cm IPG strip of pH 3–10 nonlinear (GE Healthcare) was used for first dimension electrophoresis and 250 µg of protein extract was loaded on the strip. Rehydration was followed by electrophoresis at 500 V for 2 h, 1000 V for 1 h, and 8000 V for 3 h. After equilibration the strips were separated on 12% SDS gel for 3 h at 150 volts. Silver-stained gels were scanned using an UMAX PowerLook 2100XL Image Scanner (GE Healthcare). Image spots were detected, matched, and manually edited with the Image Master-2D Platinum 6.0 software (GE Healthcare). Three independent 2D gels per treatment were imaged, analyzed and compared. Quantification was given as a spot volume percentage (vol %), with each single spot volume normalized with respect to the total spot volume of the 2-DE gel. The differences in spot volume was used as a measure of changes in protein expression levels between the treated and control groups and spots with significantly different intensities (p<0.05) were selected for further analyses.

### Mass spectrometry and database search

Protein spots showing more than 5-fold increase or decrease in spot intensity were excised, destained, and digested as described previously [44]. The digested proteins were extracted and purified with reversed-phased C18 ZipTips (Millipore ZTC18S096). Matrix-assisted laser desorption ionization time-of-flight mass spectrometry (MALDI-TOF MS) with a Bruker AutoflexII TOF/TOF mass spectrometer (Bruker Daltonics) was carried out as described by Wang et al. The peptide mass data were searched against NCBInr database with the Mascot search engine (Matrix Science Ltd., UK, http://www.matrixscience.com/) and with *Homo sapiens* as the species searched. The following settings were used for the identification: two missed cleavage sites were allowed, cysteine was carbamidomethylated and methionine was allowed to be partially oxidized.

### Protein networks and classification

To construct a protein interaction map, we used the STRING 8.2 software (http://string-db.org/) which predicts protein-protein interactions, including both physical and functional interactions [45]. It weights and integrates information from numerous sources, including experimental repositories, computational prediction methods and public text collections. Differentially expressed proteins were classified according to their main biological processes as well as their molecular functions using Swiss-Prot (http://www.uniprot.org/).

### Statistical analysis

All data were derived from at least three independent experiments. Statistical analyses were conducted using Graphpad Prism software and values were presented as mean ± SD. Significant differences between the groups were determined by Student's t tes. Data in fig. 2 and 3 was analysed by ANOVA followed by tukey's multiple comparison test (GraphPad Prism5). A value of p<0.05 was accepted as an indication of statistical significance.

## References

[1] Griffin DE, Knipe DM, Howley PM, Griffin DE, Lamb RA, Martin MA, Roizman B, Straus SE (2001). Measles virus.. Fields Virology.

[2] Egelman EH, Wu SS, Amrein M, Portner A, Murti G (1989). The Sendai virus nucleocapsid exists in at least four different helical states.. J Virol.

[3] Finch JT, Gibbs AJ (1970). Observations on the structure of the nucleocapsids of some paramyxoviruses.. J Gen Virol.

[4] Lamb RA, Kolakofsky D, Fields BN, Knipe DM, Howley PM (2001). Paramyxoviridae: The viruses and their replication.. Fields Virology.

[5] Longhi S, Receveur-Brechot V, Karlin D, Johansson K, Darbon H (2001). The C-terminal domain of the measles virus nucleoprotein is intrinsically disordered and folds upon binding to the C-terminal moiety of the phosphoprotein.. J Biol Chem.

[6] Bourhis JM, Johansson K, Receveur-Brechot V, Oldfield CJ, Dunker KA (2004). The C-terminal domain of measles virus nucleoprotein belongs to the class of intrinsically disordered proteins that fold upon binding to their physiological partner.. Virus Res.

[7] Bourhis JM, Receveur-Brechot V, Oglesbee M, Zhang X, Buccellato M (2005). The intrinsically disordered C-terminal domain of the measles virus nucleoprotein interacts with the C-terminal domain of the phosphoprotein via two distinct sites and remains predominantly unfolded.. Protein Sci.

[8] Johansson K, Bourhis JM, Campanacci V, Cambillau C, Canard B (2003). Crystal structure of the measles virus phosphoprotein domain responsible for the induced folding of the C-terminal domain of the nucleoprotein.. J Biol Chem.

[9] Zhang X, Glendening C, Linke H, Parks CL, Brooks C (2002). Identification and characterization of a regulatory domain on the carboxyl terminus of the measles virus nucleocapsid protein.. J Virol.

[10] Zhang X, Bourhis JM, Longhi S, Carsillo T, Buccellato M (2005). Hsp72 recognizes a P binding motif in the measles virus N protein C-terminus.. Virology.

[11] tenOever BR, Servant MJ, Grandvaux N, Lin R, Hiscott J (2002). Recognition of the measles virus nucleocapsid as a mechanism of IRF-3 activation.. J Virol.

[12] Sato H, Masuda M, Kanai M, Tsukiyama-Kohara K, Yoneda M (2007). Measles Virus N Protein Inhibits Host Translation by Binding to eIF3-p40.. J Virol.

[13] Bhella D, Ralph A, Murphy LB, Yeo RP (2002). Significant differences in nucleocapsid morphology within the Paramyxoviridae.. J Gen Virol.

[14] Fooks AR, Stephenson JR, Warnes A, Dowsett AB, Rima BK (1993). Measles virus nucleocapsid protein expressed in insect cells assembles into nucleocapsid-like structures.. J Gen Virol.

[15] Karlin D, Longhi S, Canard B (2002). Substitution of two residues in the measles virus nucleoprotein results in an impaired self-association.. Virology.

[16] Spehner D, Kirn A, Drillien R (1991). Assembly of Nucleocapsid like structures in animal cells infected with a vaccinia virus recombinant encoding the measles virus nucleoprotein.. J Virol.

[17] Warnes A, Fooks AR, Dowsett AB, Wilkinson GW, Stephenson JR (1995). Expression of the measles virus nucleoprotein gene in Escherichia coli and assembly of nucleocapsid-like structures.. Gene.

[18] Nicoletti I, Migliorati G, Pagliacci MC, Grignani F, Riccardi C (1991). A rapid and simple method for measuring thymocyte apoptosis by propidium iodide staining and flow cytometry.. J Immunol Meth.

[19] Thornberry NA, Lazebnik Y (1998). Caspases: enemies within.. Science.

[20] Kerr JF, Wyllie AH, Currie AR (1972). Apoptosis: a basic biological phenomenon with wide-ranging implications in tissue kinetics.. Br J Cancer.

[21] Kerr JF (1965). A histochemical study of hypertrophy and ischaemic injury of rat liver with special reference to changes in lysosomes.. J Pathol Bacterio.

[22] Singh NP, Pfeifer J (1996). Microgel electrophoresis of DNA from individual cells: principles and methodology.. Technology for Detection of DNA Damage and Mutation.

[23] Chua BT, Volbracht C, Tan KO, Li R, Yu VC (2003). Mitochondrial translocation of cofilin is an early step in apoptosis induction.. Nat Cell Biol.

[24] Klamt F, Zdanov S, Levine RL, Pariser A, Zhang Y (2009). Oxidant-induced apoptosis is mediated by oxidation of the actin-regulatory protein cofilin.. Nat Cell Biol.

[25] Müller N, Avota E, Schneider-Schaulies J, Harms H, Krohne G (2006). Measles virus contact with T cells impedes cytoskeletal remodeling associated with spreading, polarization, and CD3 clustering.. Traffic.

[26] Li AL, Li HY, Jin BF, Ye QN, Zhou T (2004). A novel eIF5A complex functions as a regulator of p53 and p53-dependent apoptosis.. J Biol Chem.

[27] Taylor CA, Sun Z, Cliche DO, Ming H, Eshaque B (2007). Eukaryotic translation initiation factor 5A induces apoptosis in colon cancer cells and associates with the nucleus in response to tumour necrosis factor alpha signalling.. Exp Cell Res.

[28] Zheng L, Roeder RG, Luo Y (2003). S phase activation of the histone H2B promoter by OCA-S, a coactivator complex that contains GAPDH as a key component.. Cell.

[29] Hara MR, Agrawal N, Kim SF, Cascio MB, Fujimuro M (2005). S-nitrosylated GAPDH initiates apoptotic cell death by nuclear translocation following Siah1 binding.. Nat Cell Biol.

[30] Hara MR, Thomas B, Cascio MB, Bae BI, Hester LD (2006). Neuroprotection by pharmacologic blockade of the GAPDH death cascade.. Proc Natl Acad Sci U S A.

[31] Ralser M, Wamelink MM, Kowald A, Gerisch B, Heeren G (2007). Dynamic rerouting of the carbohydrate flux is key to counteracting oxidative stress.. J Biol.

[32] Jaattela M (1999). Escaping cell death: survival proteins in cancer.. Exp Cell Res.

[33] Beere HM, Wolf BB, Cain K, Mosser DD, Mahboubi A (2000). Heat-shock protein 70 inhibits apoptosis by preventing recruitment of procaspase-9 to the Apaf-1 apoptosome.. Nat Cell Biol.

[34] Taira T, Saito Y, Niki T, Iguchi-Ariga SM, Takahashi K, Ariga H (2004). DJ-1 has a role in antioxidative stress to prevent cell death.. EMBO Rep.

[35] Kim RH, Smith PD, Aleyasin H, Hayley S, Mount MP (2005). Hypersensitivity of DJ-1-deficient mice to 1-methyl-4-phenyl-1,2,3,6-tetrahydropyrindine (MPTP) and oxidative stress.. Proc Natl Acad Sci U S A.

[36] Wang S, Nath N, Adlam M, Chellappan S (1999). Prohibitin, a potential tumor suppressor, interacts with RB and regulates E2F function.. Oncogene.

[37] Wang S, Nath N, Fusaro G, Chellappan S (1999). Rb and prohibitin target distinct regions of E2F1 for repression and respond to different upstream signals.. Mol Cell Biol.

[38] Phillips AC, Vousden KH (2001). E2F-1 induced apoptosis.. Apoptosis.

[39] Watanabe M, Hirano A, Stenglein S, Nelson J, Thomas G (1995). Engineered serine protease inhibitor prevents furin-catalyzed activation of the fusion glycoprotein and production of infectious measles virus.. J Virol.

[40] Oh KS, Engler JA, Joung I (2005). Enhancement of gene delivery to cancer cells by a retargeted adenovirus.. J Microbiol.

[41] Wickham TJ (2000). Targeting adenovirus.. Gene Ther.

[42] Hishikawa K, Oemar BS, Tanner FC, Nakaki T, Luscher TF (1999). Connective tissue growth factor induces apoptosis in human breast cancer cell line MCF-7.. J Biol Chem.

[43] Tice RR, Agurell E, Anderson D, Burlinson B, Hartmann A (2000). Single cell gel/comet assay: guidelines for in vitro and in vivo genetic toxicology testing.. Environ Mol Mutagen.

[44] Wang Z, Yip C, Ying Y, Wang J, Meng XY (2003). Mass spectrometric analysis of protein markers for ovarian cancer.. Clin Chem.

[45] Jensen LJ, Kuhn M, Stark M, Chaffron S, Creevey C (2009). Nucleic Acids Res.

